# SBAS-InSAR based validated landslide susceptibility mapping along the Karakoram Highway: a case study of Gilgit-Baltistan, Pakistan

**DOI:** 10.1038/s41598-023-30009-z

**Published:** 2023-02-27

**Authors:** Isma Kulsoom, Weihua Hua, Sadaqat Hussain, Qihao Chen, Garee Khan, Dai Shihao

**Affiliations:** 1 School of Geography and Information Engineering, China University of Geosciences (Wuhan), Wuhan, 430074 China; 2 Department of Geological Engineering, University of Engineering and Technology, (Lahore), Lahore, 54890 Pakistan; 3 Department of Earth Sciences, Karakoram International University, Gilgit, 15100 Pakistan

**Keywords:** Climate sciences, Environmental sciences, Natural hazards

## Abstract

Geological settings of the Karakoram Highway (KKH) increase the risk of natural disasters, threatening its regular operations. Predicting landslides along the KKH is challenging due to limitations in techniques, a challenging environment, and data availability issues. This study uses machine learning (ML) models and a landslide inventory to evaluate the relationship between landslide events and their causative factors. For this, Extreme Gradient Boosting (XGBoost), Random Forest (RF), Artificial Neural Network (ANN), Naive Bayes (NB), and K Nearest Neighbor (KNN) models were used. A total of 303 landslide points were used to create an inventory, with 70% for training and 30% for testing. Susceptibility mapping used Fourteen landslide causative factors. The area under the curve (AUC) of a receiver operating characteristic (ROC) is employed to compare the accuracy of the models. The deformation of generated models in susceptible regions was evaluated using SBAS-InSAR (Small-Baseline subset-Interferometric Synthetic Aperture Radar) technique. The sensitive regions of the models showed elevated line-of-sight (LOS) deformation velocity. The XGBoost technique produces a superior Landslide Susceptibility map (LSM) for the region with the integration of SBAS-InSAR findings. This improved LSM offers predictive modeling for disaster mitigation and gives a theoretical direction for the regular management of KKH.

## Introduction

**Figure 1:**
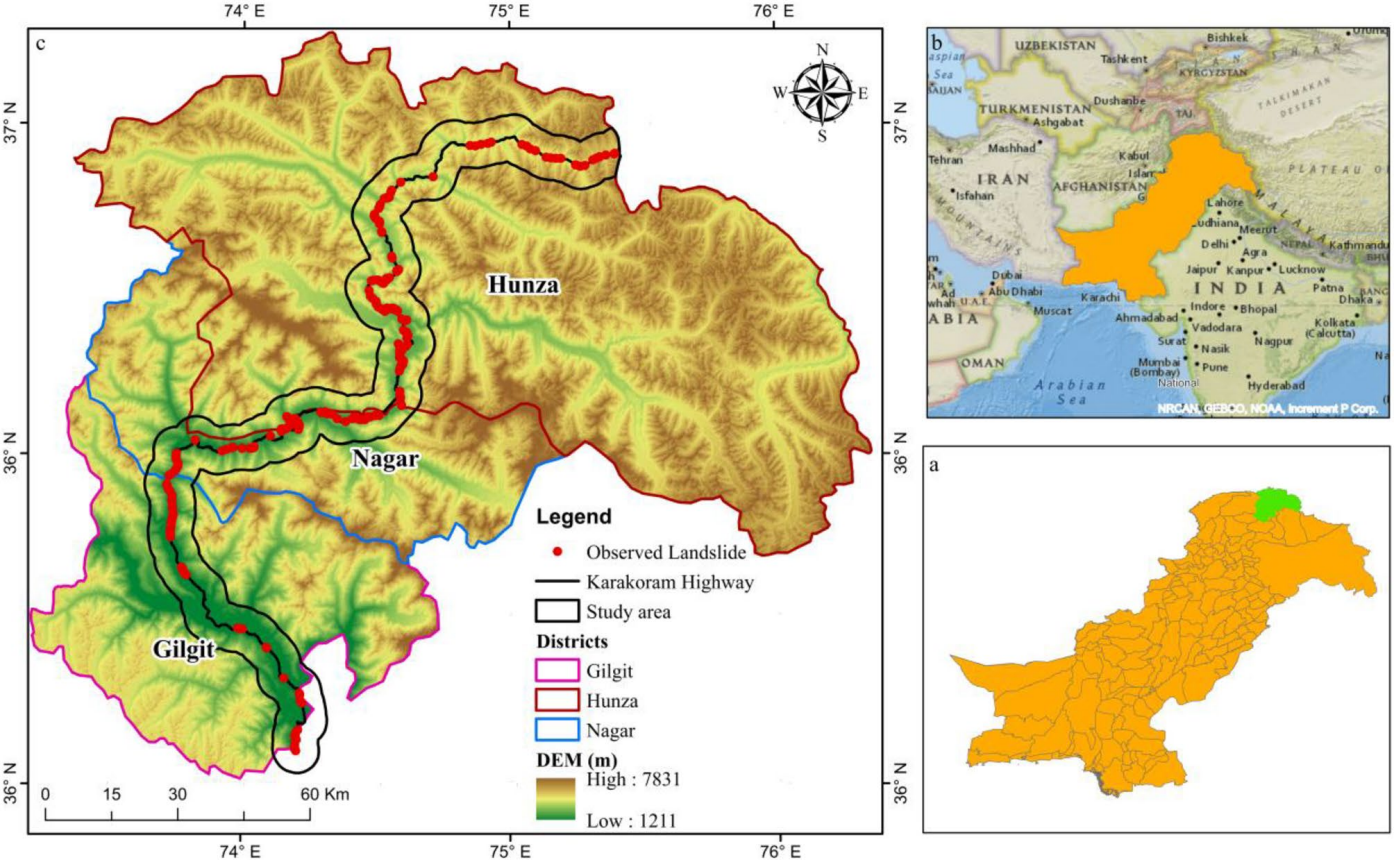
A map showing the study region; (**a**) Pakistan's geographic map representing district boundaries; (**b**) a map of Pakistan showing its geographical boundaries; and (**c**) a Digital Elevation Model of Gilgit Baltistan where points show Landslides of the study region, and the black line shows the KKH.

Landslides are major geological hazards in terms of human and property loss. They occur when gravitational forces cause rock, debris, or earth shear resistance to fail^1,2^. The mountainous terrains of Pakistan's Gilgit Baltistan (GB) province are prone to landslides due to earthquakes, snowmelt, heavy rains, land usage changes, and other human activities. These mountains have a reputation for geological instability, with reports of rock falls, rockslides, avalanches, rotating slips, slumps, debris flow, and creep^3^. The Karakoram Highway (KKH) in Pakistan's GB province is a high-elevation, paved highway that connects Pakistan and China's Xinjiang region. It is the only trade route between the two countries and has become increasingly important to their economies due to the China-Pakistan Economic Corridor (CPEC) initiative. KKH is often called the "Eighth Wonder of the World" (UNESCO 2010) due to its construction in challenging circumstances^4^. Hundreds of rockfalls, rockslides, and debris flow along the KKH have damaged its reputation since it was finished in 1979^5,6^. In 2010, Attaabad Lake was formed when a landslide blocked the Hunza River, burying 19 km of KKH and causing the deaths of 20 people and the destruction of 350 homes^5,6^. Since 2011, researchers have investigated 150 glacier debris flows that have caused damage to road bridges and blocked transportation on KKH^7–9^. This study investigated and compiled a landslide inventory of over 332 km of roadway in the Gilgit, Nagar, and Hunza districts Fig. 1. The KKH has brittle rocks, varied climates, topography, shifting stratigraphy, and varying tectonic activity. Given these factors, the area has been classified as a geohazard natural laboratory for scientific study, and the Landslide Susceptibility map (LSM) is crucial in assessing hazards and developing plans for high-risk areas^10,11^.

Remote sensing (RS) and Geographic Information Systems (GIS) in LSM have been recommended by researchers^2,12,13^ as effective methods for generating a landslide inventory by evaluating and assessing the possibility of landslide occurrences in landslide-prone regions. Landslide Causative Factors (LCFs) are a database of geospatial attributes that may affect slope stability in landslide regions, including elevation, slope angle, precipitation, TWI, and lithology. This database is constructed using GIS data sources. The LCFs data can be employed to model the response of additional slopes and predict future landslides in study region^2^. This work created an LSM along the KKH using a database of fourteen LCFs.

Due to the intricate nature of landslide hazards, numerous physical and statistical models have been developed for LSM^14,15^. It has been shown that each method has its benefits and limitations^16,17^. For example, Physical models provide reliable forecast accuracy and are useful for localized mapping and sub-catchment analysis but require detailed site characterization. Surface data and subsurface monitoring methods are necessary for predicting slope failures^15,18^. However, Physical models require huge amounts of accurate data for reliable outcomes, which can be costly for large-scale studies. Consequently, physical-based models cannot be used for large-scale hazard zonation. However, statistical models, aided by GIS advancements, have numerous quantitative approaches and techniques for modeling landslides that improve the interpretation of patterns and generating processes^15,19^. Many landslide susceptibility models have been developed using various statistical methods in Machine Learning over the past two decades for accurate results. Machine learning models are useful for addressing nonlinear geospatial issues due to geological, geotechnical, and climatic variables.

LSM has improved recently due to improvements in ML and geospatial technology^15,20,21^. Nowadays, LSM with high precision can be evaluated by identifying the relationship between LCFs and slope instability with advanced ML methods^22^. Many researchers have used various ML models, including logistic regression (LR)^23,24^, boosted regression tree^25^, support vector machine (SVM)^26–28^, artificial neural network (ANN)^26,27^, naïve bayes (NB)^29^, maximum entropy (maxENT)^30^, extreme gradient boosting (XGBoost)^3^, to predict landslides. Merghadi et al.^15^, comprehensively analyze the structure and working mechanism of the most popular ML algorithms. Numerous attempts have been undertaken to execute, explore, and assess these ML approaches in various geographic settings^20,31–33^. For instance, Merghadi et al.^15^, examined the performance and prediction capability of random forest (RF), SVM, gradient boost machine (GBM), LR and ANN in the Mila basin, Algeria. According to their findings, GBM and RF outperformed the other ML algorithms with AUCs of 0.897 and 0.895, respectively. Wang et al.^34^, did a similar evaluation in the terrains of Shexian County, China, for LSM using various ML models coupled with GIS tools. The results of this investigation showed that the SVM and RF models achieved the best outcomes with AUCs of 0.821 and 0.803, respectively. Several ML models, such as SVM, generalized linear models (GLM), NB, and other tree-based models, were recently deployed by Qing et al.^35^, to investigate the vulnerability regarding a debris flow along the China-Pakistan Karakoram Highway. The authors tested many distinct modelling approaches according to watershed and catchment limits around the highway's periphery and discovered that the SVM performed best using an AUC of 0.96. Pham et al.^29^, assessed the LSM of Uttarkhand, India, using five ML models, and performance was assessed by the ROC curve and statistical Index based method. According to the results, all models performed well; however SVM model outperforms the other landslide models with an AUC of 0.922. As a conclusion to existing research, we may conclude that the accuracy of ML models in LSM relies on training data that includes geological settings, topography, climate and dataset of historical landslides in the area. There is "no rule of thumb" regarding which ML method is appropriate for LSM due to the high-level degree of uncertainty and diverse topographical and environmental factors of locations^36^. Examining the dynamics of landslides and susceptibility for appropriate risk management and planning is crucial to testing these algorithms under different geographic settings.

Remote sensing (RS) methods can map regions with recurring large landslides. RS techniques can reduce the misclassification of LSM and provide a solution in the form of enhanced detection and surveys^37^. Interferometric synthetic aperture radar (InSAR) techniques for radar images are a powerful tool for huge landslide mapping and identification, which might support the appraising and building landslide inventory maps. InSAR techniques are ideal for slow linear and nonlinear deformation of prolonged sequences, as mentioned in^38,39^.

Previous studies^5,40–42^ in the area have emphasized analyzing the quantitative and deterministic links and regression analysis between landslides with causative factors. These traditional statistical methods cannot correctly map and predict landslide hazards. Also, researchers have employed ML and RS methods separately for providing an LSM. Therefore, there is an unknown gap in understanding the suitable techniques for LSM. In this regard, this study employed XGBoost, RF, ANN, NB, and KNN with the SBAS-InSAR technique as evident methods for evaluating LSM. The model with the best accuracy is validated by the SBAS-InSAR technique and survey data, making it a more effective, novel method for identifying surface deformations. In high-risk areas, SBAS-InSAR can locate and characterise individual landslides. Multiple time series of synthetic aperture radar (SAR) imageries may be evaluated to determine the velocity of a landslide using spatial statistical techniques.

This research aimed to use ML models such as XGBoost, RF, ANN, KNN, and NB to build a susceptibility map and compile a comprehensive, visually interpreted inventory of landslides. These cutting-edge ML models can quantify regional environmental problems and risks. The second objective was to employ SBAS-InSAR to assess high-risk areas for future landslide risk reduction by estimating slow-moving landslides' deformation rates. The final objective was to use SBAS-InSAR findings and field survey data to develop a new LSM for the region, with the best susceptibility model determined based on accuracy and AUC value. These projections will also guide regional and global scale for land use development and may reduce human and economic costs along this crucial highway.

## Materials and methods

### Study area

The study was directed along the KKH, which passes through the districts of Gilgit, Hunza, and Nagar of Gilgit Baltistan, Pakistan. This research focuses on a significant section of the KKH, which has a total length of 332 km and includes a 10 km buffer zone (Fig. 1). The study region covers an area of 3320 km^2^. The research region consists of a chain of villages through which the KKH passes, beginning with Juglot, and ending with Khunjarab top, the China–Pakistan border checkpoint. The region's terrains are rough, ranging from 1211 to 7831 metres above mean sea level. Structurally, the region is complex because it lies in the subduction zone (Main Karakoram Thrust).

Moreover, the mountains have steep slopes that are prone to landslides^43^. The research region's most common landslides are debris, and rock falls induced by precipitation and seismic activity^6^. Most of the rocks are Mesozoic and Paleozoic in age. The majority of the region's exposed rocks are volcanic, volcano-sedimentary, metamorphic, sedimentary, and igneous. These rocks are divided into siliciclastic, basalt, carbonates, andesite, gabbro, granite, greenschist and so on.

Gilgit Baltistan has around 154 mm of rain each year. Water irrigation for land cultivation is supplied by rivers and streams overflowing with snowmelt and glacial water from mountainous regions. Summer is more prolonged, drier, and hotter. Strong sunlight occasionally elevates temperatures beyond 40 °C (104 °F), although the winter's average temperature remains below 10 °C. There are numerous landslides and avalanches in the region due to the harsh weather conditions^44^. The region's geological traits and soils, which also play a crucial role, are fragile.

### Landslide data and inventory

**Figure 2:**
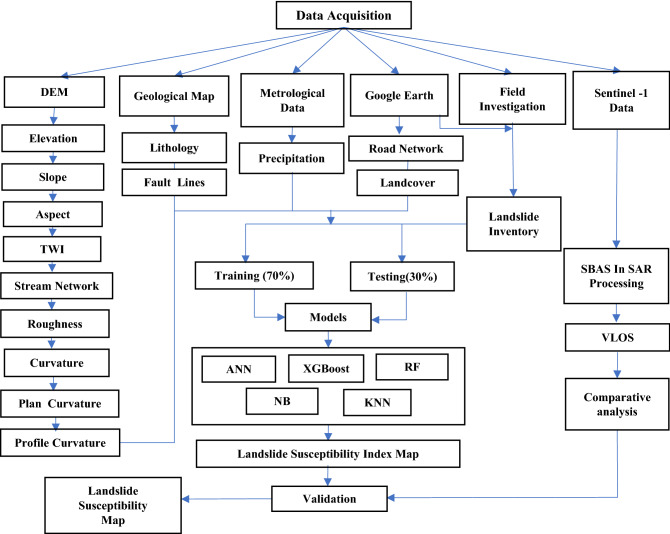
The research framework of the study.

The data for this study consisted of a 30m SRTM DEM, a geology map of the Pakistan geological survey scale (1:50,000), sentinel-2 images 10m, and meteorological data 30m. The factors evaluated for LSM along the KKH were slope, elevation, curvature, aspect, profile curvature, plan curvature, Roughness, Topographic Wetness Index (TWI), and proximity to stream derived from the DEM. Landcover derived from sentinel-2 images, annual precipitation derived from metrological data, proximity to road derived from google earth and surface lithology, and proximity to fault data derived from the geological map in the ArcGIS environment. Twenty-four ascending and twenty-three descending Sentinel-1A images were obtained for SBAS-InSAR processing to evaluate the displacement velocity. Fig. 2 illustrates the overall procedure.

There were found a total of 303 landslide points along the KKH using remote sensing image analysis, geological maps, survey data, meteorological data, and historical data collection^45^. These were obtained from various sources for the research. The inventory of landslides was developed by visually interpreting Sentinel 2 images, which were then cross-checked by Google Earth imagery, field data, and the SBAS-InSAR technique. The significant effect of each observed landslide during fieldwork was shown on a proper scale; topographic maps were then digitized as a polygon layer^46,47^. For the inventory, 303 landslide points were mapped in the research region. It provides information about each landslide's location, magnitude, and direction in the inventory, bedrock, and surface material. The inventory was split into training (70%) and testing (30%) sets for constructing Landslides Susceptibility Mapping. Table 1 lists the datasets that were used.

**Table 1:** The components, extraction, and categorization of input parameters.

Variables	Description/Extraction	Category
Slope	DEM	Geomorphology
Aspect	DEM	Topography
Elevation	DEM	Topography
Curvature	DEM	Geomorphology
Plain curvature	DEM	Geomorphology
Profile curvature	DEM	Geomorphology
Roughness	DEM	Geomorphology
Proximity to stream	DEM	Hydrology
TWI	DEM	Geomorphology
Proximity to fault	Geology	Geology
Lithology	Surface Lithology	Geology
Landcover	Landcover classes	Land use
Proximity to road	Google Earth	Topography
precipitation	Annual rainfall	Climate factor

### Landslide causative factors

**Figure 3:**
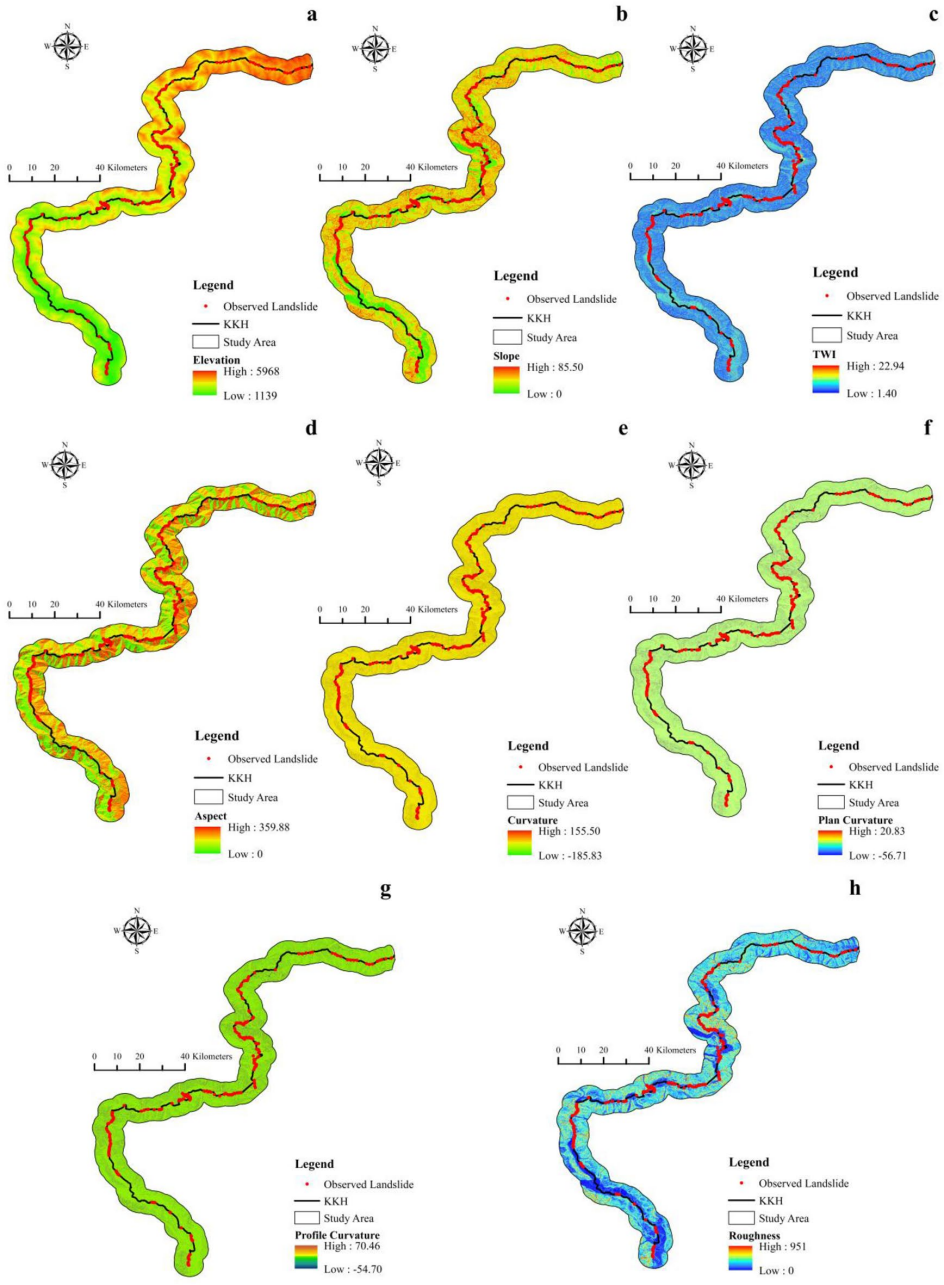
Landslide components. (**a**) elevation, (**b**) slope, (**c**) TWI, (**d**) aspect, (**e**) curvature, (**f**) plan curvature, (**g**) profile curvature, (**h**) roughness.

**Figure 4:**
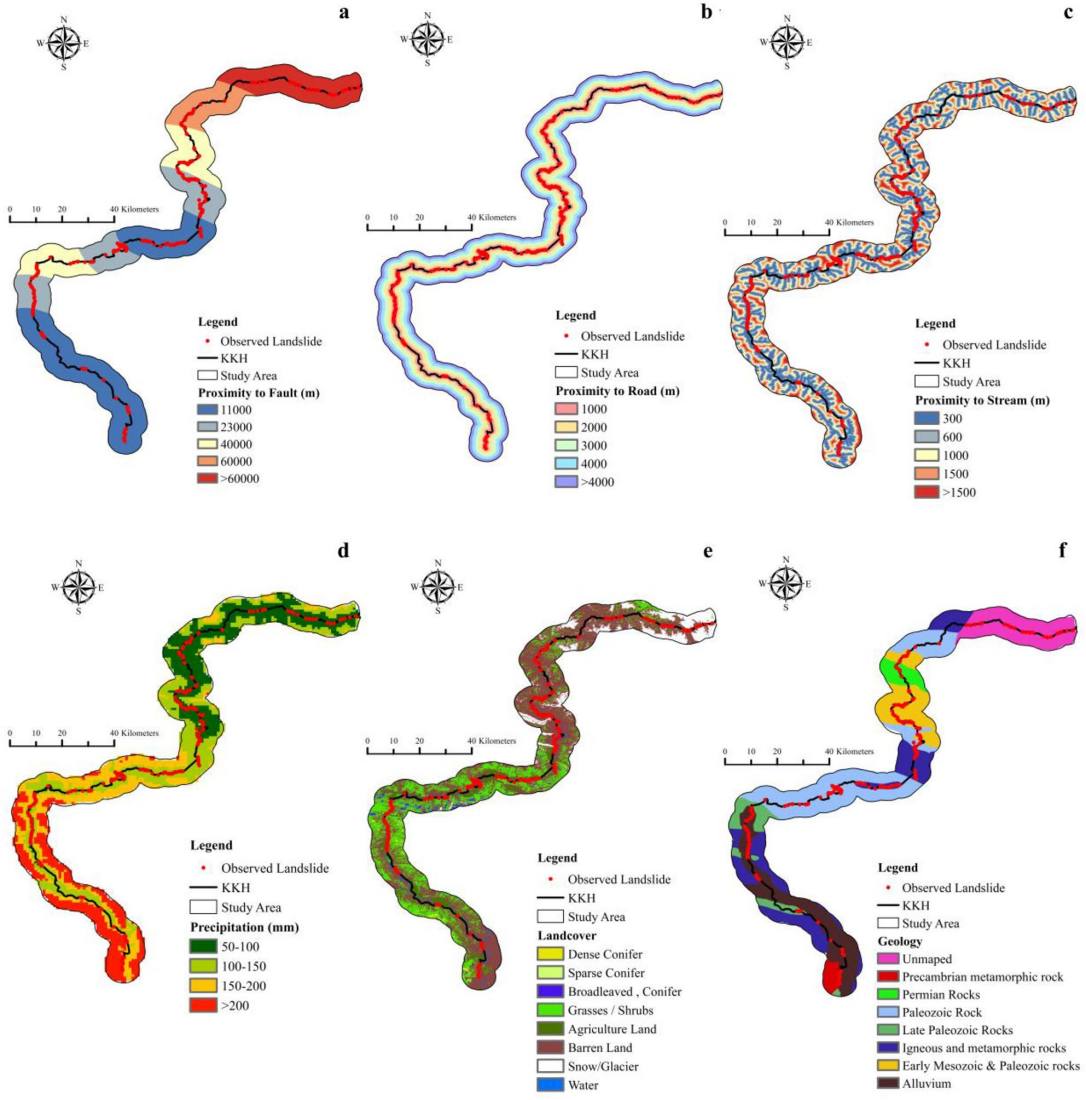
Landslide components. (**a**) proximity to fault, (**b**) proximity to road, (**c**) proximity to stream, (**d**) precipitation, (**e**) landcover, (**f**) geology.

GIS tools are extensively employed to extract crucial susceptibility evaluation elements from digital elevation models (DEM), including slope, aspect, elevation, and roughness. Lithology, precipitation, land cover, plan curvature, aspect, Topographic Wetness Index (TWI), slope, elevation, proximity to road, proximity to fault, profile curvature, roughness, proximity to a stream, and curvature are used to determine the probability of landslide fatalities across the section of KKH (Table 1). The 14 LCFs are displayed in Figs. 3 and 4.

The modeling method includes identifying ML models, model development and model fitting. The grid unit served as the study's model unit. The spatial resolution of remote sensing data and DEM was 30 m, and all assessment factors were resampled at this level. A condition attribute generated a two-dimensional table including 14 assessment criteria and a landslide decision characterizes (0 indicates no landslide, 1 indicates landslide), with every line indicating an object. Each column signifies an attribute of the object and is updated to train (70%) and test the two-dimensional table (30%). Training data was employed to develop the model, while test data were employed to obtain predictions. Model units in the research region were computed using the five ML models mentioned above. The Landslide susceptibility Index (LSI) maps were generated using model prediction values. The five ML model results were imported into the GIS, and LSM was generated. LSM was then separated into five classes using the Janks natural breakpoint^48^ named very low, low, moderate, high, and very high. The five ML models were carefully examined utilizing the area under the ROC curve.

### Landslide susceptibility models

The Landslide Susceptibility map for ANN, XGBoost, RF, NB, and KNN was prepared using the R programming language.

#### Artificial neural network (ANN)

An artificial neural network (ANN) is a compilation of linked connections used to represent issues with the complex relationship between several assessment variables^49^. Because of its dynamic and nonlinear nature, ANN is considered suitable for landslide susceptibility studies. ANN algorithms improve the extraction of extensive relationships between the different independent landslide factors^50^. A neural network comprises several artificial neural connections that may be used to estimate or approximate functions. ANN is typically composed of two layers of input (conditioning factor) and a set of secondary layers (hidden layer); that execute output layers, with the predicted results performed by utilizing hidden layers.

The aim of an ANN is to develop a model of the data-generation process so that the network can comprehend and predict outcomes from inputs that have never been seen before^51^. The "NNET" program was utilized in this study to carry out an ANN model with a 14-2-1 network. Table 2 lists the primary parameters which are used in ANN.

**Table 2:** Hyperparameters of XGBoost, RF, NB, and ANN.

Models	Parameters	Values
XGBoost	Max_depth	6
	nrounds	200
	eta	0.05
	colsample_bytree	0.75
	subsample	1
ANN	Hidden layers	2
	Loss function	Cross entropy
	stepmax	1e = 08
R.F	Seed	1234
	nodesize	14
	ntree	500
	mtry	5
NB	Nround	210
	fL	0
	usekernel	T
	adjust	1.0

#### Extreme Gradient Boosting (XGBoost)

The XGBoost supervised classification model is created on the Gradient tree boosting algorithm^52,53^, an effective ML method developed by Chen and Guestrin (2016). XGBoost is designed to train with multiple Processing cores, and it can identify and learn upon nonlinear data patterns; regularized boosting is employed to reduce overfitting and increase model precision, making it more efficient than over-boosting techniques^54,55^. XGBoost provides scalability for many use cases with low computational resource requirements, good performance (i.e., speed), handling of sparse data, and ease of implementation^56^. Training in XGBoost is done using an additive technique, which was also awarded as the winner of numerous data science contests. Model XGBoost involves numerous model preview settings to be selected. Three primary hyperparameter settings are necessary for model training: nrounds (maximum number of boosting iterations), subsamples (the training instance subsample ratio) and colsample bytree (columns ratios sub-sample when each tree is formed) (Table 2).

#### Random Forest (RF)

In classification and regression, random forest is employed. It employs the majority vote for categorization and the average for regression from numerous samples^57^. RF can handle both continuous and categorical variables in regression and classification. It outperforms other categorization algorithms^58^. The primary problem with this approach is that the results of each tree differ from each other^59^. A random forest strategy is offered to reduce these variances and change approximation^60^. It incorporates several decision trees that employ several data-driven base classifiers, and several parameters are selected randomly to develop an individual tree^61^. Table 2 lists the three most important hyperparameters: the number of features that are suitable for division (mtry), The minimal amount of samples that are randomly selected for each random subset to achieve tree balance using recursive portioning., and the number of bootstrap samples to employ (ntree).

#### Naïve Bias (NB)

The NB model is a method for supervised learning that employs the Bayes theorem to overcome classification problems^62^. The NB Classifier is a basic and efficient classification technique that promotes the development of robust ML models by generating immediate forecasts^63^. It is a predictive model that makes predictions based on the likelihood of an object. It is assumed that the significance of a particular attribute is independent of the occurrence of other characteristics^64^. For example, if the landslide is identified based on causative variables, the landslide is recognized as a catastrophe. As a result, each feature contributes to evaluate if it is a hazard without depending on the others. Many studies have used the NB approach to map landslide susceptibility^29,65^. Table 2 displays the parameters used in NB for this study.

#### K-Nearest Neighbor (KNN)

KNN is among the most prominent and efficient algorithms for detecting patterns in classification and regression applications^66^. It is an unsupervised method that is also known as the lazy learning algorithm^67^. It operates by determining the distance between a single test observation and all of the training dataset's observations and then locating its K nearest neighbors. This occurs with each test observation, in which common variables in the dataset are discovered^68^. KNN calculates distances by selecting a distance metric from several available metrics (e.g., Euclidean, Manhattan, etc.)^69^.

### SBAS InSAR technique

The InSAR technique has been extensively employed for the early detection of landslides because of its advantage of being weather independent and possessing a broad monitoring scope and high accuracy monitoring. The SBAS is a multi-temporal InSAR technique that uses a stack of SAR interferograms to spot slow-moving deformations with millimetre-level accuracy^70,71^. InSAR is a time series-centred technique generally categorized into two classes: the PS-InSAR approach, which works on the positions of persistent scatterers (PS), and the small baseline (SBAS) technique, which focuses on spatial connection and dispersed scattering^72,73^.

This study processed forty-seven sentinel-1A images from the year 2021 in the SARScape module. The sensor has several acquisition ways, involving wave (Wave), interferometric wide (IW), extra-wide swath (EW) and strip map (SM)). This research collected imagery from the Sentinel-1A IW sensor and used ENVI software to evaluate them (12 days of temporal resolution). As indicated in Table 3, For SBAS-InSAR processing, the line of sight (LOS) displacement velocity (VLOS) was estimated using a coherence threshold of 0.35 to prevent the consequences of unwrapping errors^74^.

**Table 3:** Details of SBAS InSAR processing.

Specifications	Ascending	Descending
Temporal range	Jan, 2021–dec, 2021	Jan, 2021–dec, 2021
No. of images	24	23
Orbit direction	Ascending	Descending
No. of cells	500,000	500,000
Minimum VLOS (mm/year)	− 120	− 114
Maximum VLOS (mm/year)	101	88

**Figure 5:**
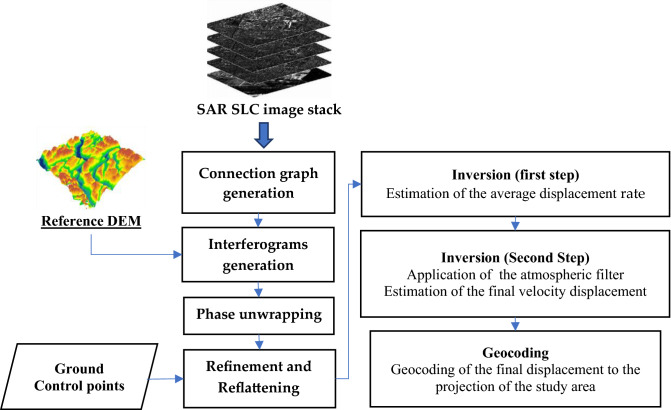
Flowchart of SBAS-InSAR.

This section uses the SBAS-InSAR technique to validate the LSM along the KKH. Figure 5 shows the fundamental data processing chart, which includes data (SAR and DEM) preprocessing, interferometric generation, phase unwrapping, refinement and reflattening estimation, and deformation calculations.

**Figure 6:**
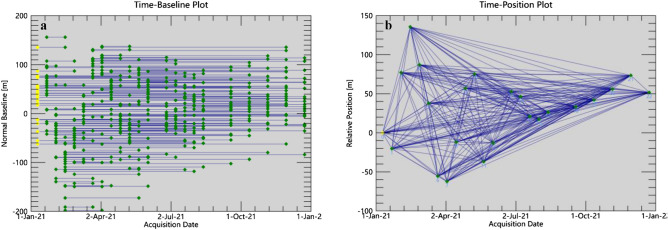
Temporal and Spatial baseline graph, The yellow dots represent the image of the super master, while the green dots represent the image of the slaves. The blue lines depict interferometric pairs. (**a**) The Time-Baseline plot; (**b**) the Time-position plot.

#### Data preprocessing

Data preprocessing includes the calculation of time and space baselines between all Sentinel-1A image pairs. After registration and clipping, the DEM data is used to complete image registration, and the relative combination that satisfies a given threshold is selected to produce a differential interferogram set^75^. This study uses a 30 m resolution SRTM DEM to generate interferograms. The super main image used is taken from the images of 23rd Dec 2021, and a total of 253 interferometric image pairs were generated. The data pairing is shown in Fig. 6.

#### Deformation calculation

Inversion is the main step of SBAS-InSAR processing, and the deformation calculation is majorly based on the analysis of inversion results. The first inversion estimates the displacement rate and residual topography, and a second unwrapping is performed to optimize the input interferogram^76^. The second inversion is based on the first inversion, using low-pass and high-pass filtering to estimate and remove the atmospheric phase, to obtain the final displacement results more accurately and finally get the deformation rate distribution in the study region through geocoding.

## Results

### Significance of landslide causative factors

**Figure 7:**
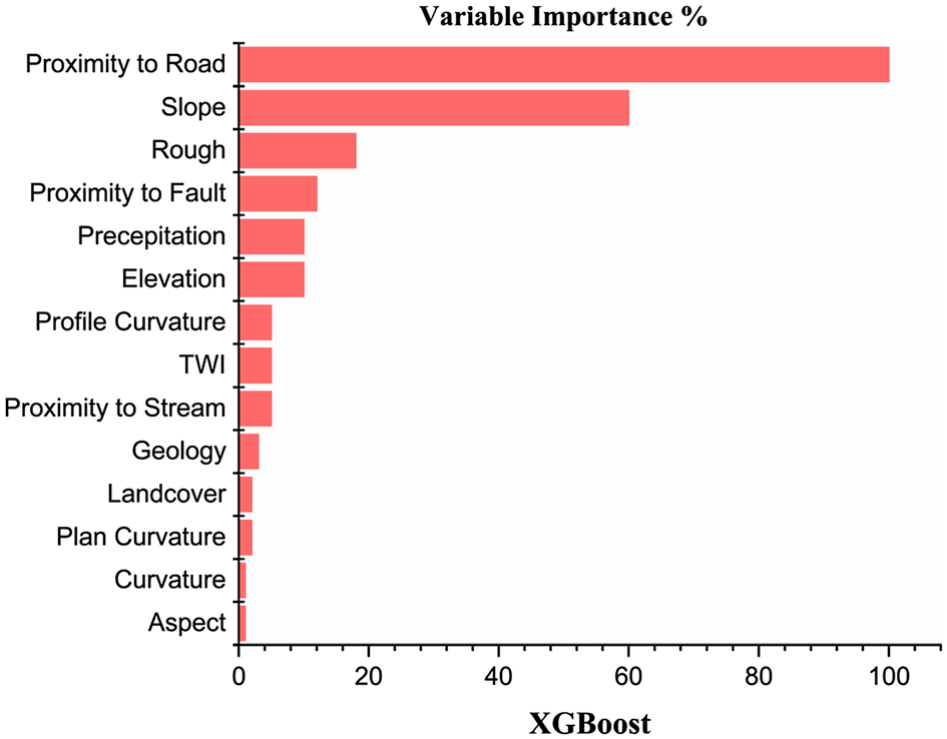
Variable’s importance in the study area.

The importance of causative factors in the occurrence of any landslide is highly significant. For this purpose, R software is used to measure the significance of each landslide element in this study. Fig. 7 demonstrates the influence of each causative factor on the landslides.

XGBoost model is employed to determine the contribution of fourteen LCFs. The outcomes (Fig. 7) show that proximity to the road, followed by slope angle, has the highest influence in initiating landslide hazards in the region because these factors directly influence the stability of slopes. A slope close to a road may be more prone to landslides due to the increased weight and vibration from vehicle traffic. Additionally, road maintenance activities, such as grading and paving, can also impact the stability of slope^77^. Other factors, i.e., roughness, proximity to the fault, precipitation and elevation, almost contribute equally to landslide occurrence. In contrast, the remaining eight factors showed the lowest contribution to landslide occurrence.

Also, The barren land is directly exposed to climatological factors such as sunlight and precipitation, which accelerates the weathering of rocks and increases the likelihood of landslides^78^. Most debris flows, rockfalls, and rock slides in region^51^ are triggered by heavy rainfall^79^. In this study, average yearly precipitation data were employed. Because yearly precipitation data can provide an overview of an area's overall wetness or dryness over a longer period, which may be useful for identifying areas that are consistently prone to landslides. High-elevation zones are frequently defined by sedimentary rock, and medium-height slopes are commonly coated with thin colluvium, thereby increasing their vulnerability to landslides^80^. The class of buffer closest to the fault is the most vulnerable. Since the area's active fault and shear zones significantly affect landslide activity (Fig. 7)^4^. The most vulnerable formations are the Yasin group and Quaternary alluvium^81^. in the research region; however, the lithological units have little impact on LSM.

### Landslide susceptibility mapping

**Figure 8:**
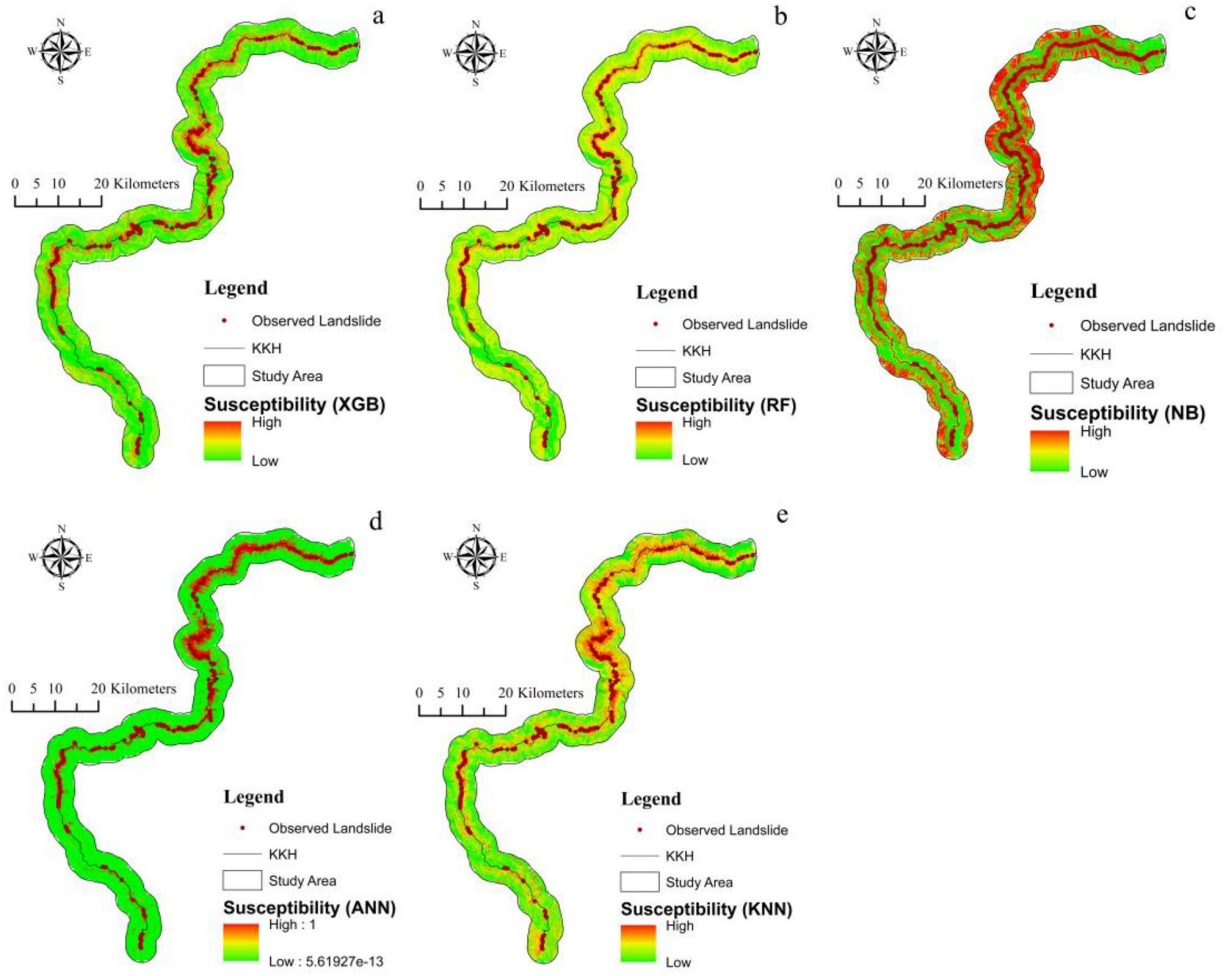
Susceptibility Index map of landslides (**a**) XGBoost, (**b**) RF, (**c**) NB, (**d**) ANN, and (**e**) KNN.

Fig. 8 shows the results of five machine learning models for LSM, identified using LSI. The higher the LSI, the higher the chance of a landslide occurring^6^. The results of ML models show that the research region is highly susceptible to landslide hazards, especially in the vicinity of Hunza, Chalt and Juglote valley. These areas are characterized by complex geological features, developed faults, and frequent earthquakes. Under the impact of sudden heavy precipitation and snow and ice meltwater, numerous landslides, rock falls, surficial instability occurrences, and complex and difficult slips, including creep, occur, badly blocking the KKH and hindering its normal operation.

**Figure 9:**
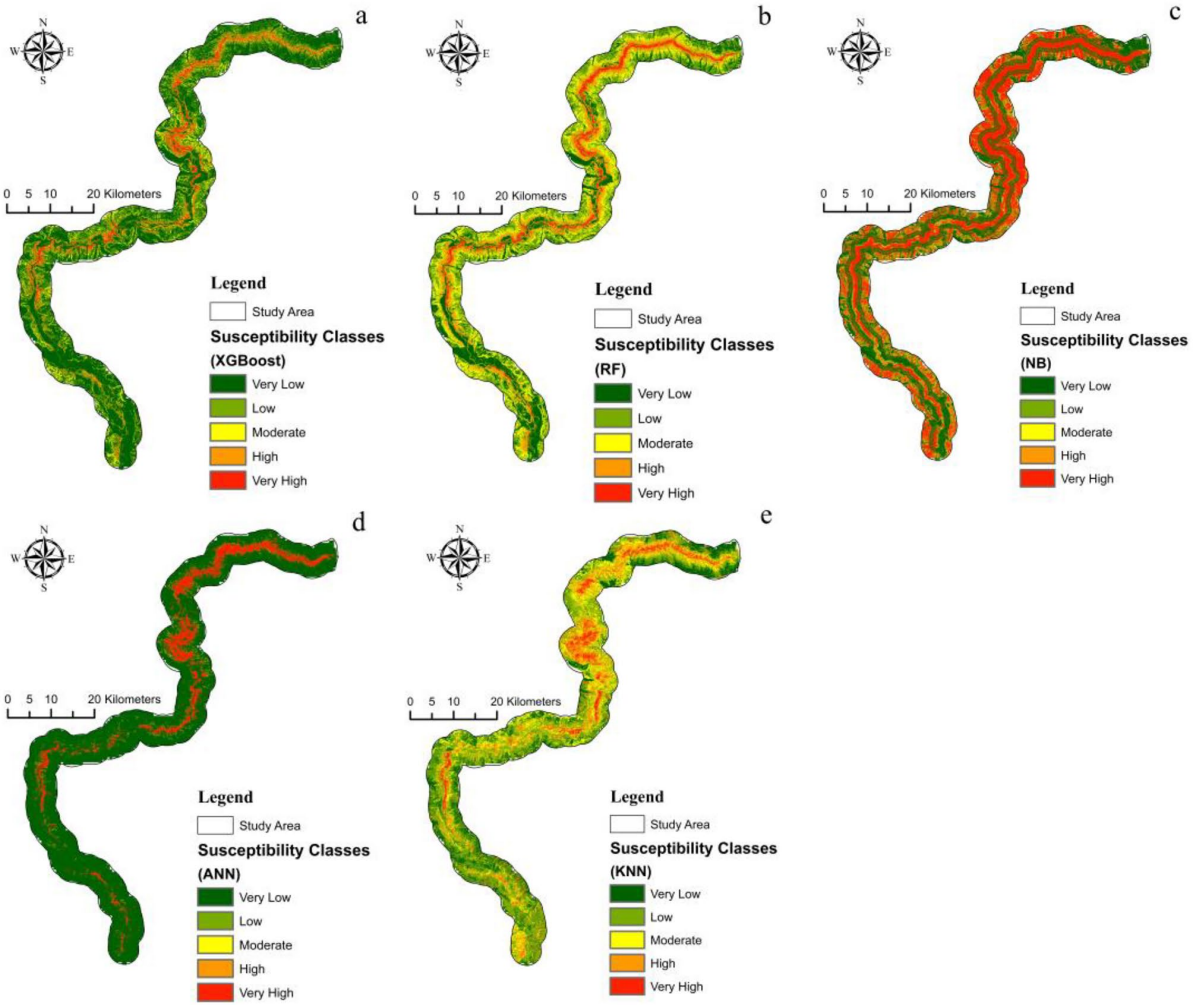
Susceptibility classes (**a**) XGBoost, (**b**) RF, (**c**) NB, (**d**) ANN, and (**e**) KNN.

The likelihood of landslide occurrence was divided into five categories using Using the natural breaks approach: very low, low, moderate, high, and very high (Fig. 9). Qualitative analysis of landslide susceptibility maps employed landslide susceptibility regions, indicating the frequency from each susceptibility level to the whole research region.

**Figure 10:**
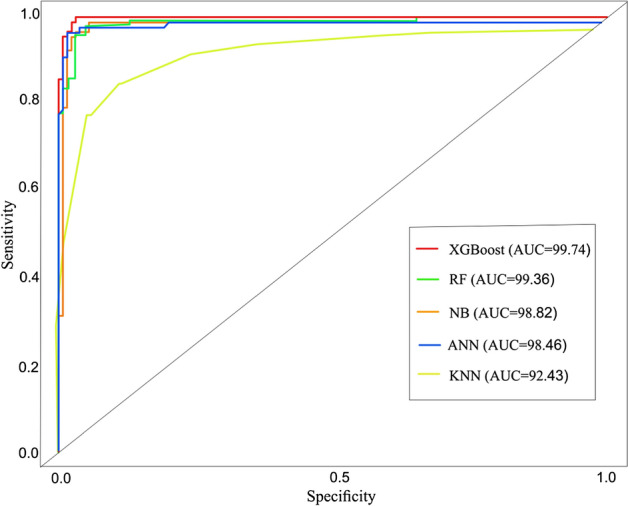
AUC plots of XGBoost, RF, NB, ANN, and KNN.

In the training phase, a confusion matrix illustrates the capabilities of the five-machine learning models. Table 4 displays the confusion matrix results for each of the five models. In the area of research, the XGBoost model has high accuracy (0.972) and AUC (0.997). Validation has been performed using the valid receiver operating characteristic (ROC) technique^82^. This method produces the ROC curve by graphing sensitivity against specificity using cutoff values; however, this does not adequately describe the model's accuracy. Consequently, The AUC of a ROC curve is utilized to evaluate the overall computational efficiency of model^83^. Based on the findings, the AUC is 99.74% for XGBoost, 99.36% for RF, 98.82% for NB, 98.46% for ANN, and 92.43% for KNN (Fig. 10).

**Table 4:** Confusion matrix XGBoost, RF, NB, ANN, and KNN.

Models	Label	Predicted Label		Accuracy
		No	Yes	
XGboost	No	86	1	0.972
	Yes	4	89	
RF	No	85	2	0.961
	Yes	5	88	
NB	No	41	1	0.890
	Yes	49	89	
ANN	No	81	9	0.884
	Yes	2	88	
KNN	No	73	8	0.861
	Yes	17	82

### SBAS- InSAR validation

**Figure 11:**
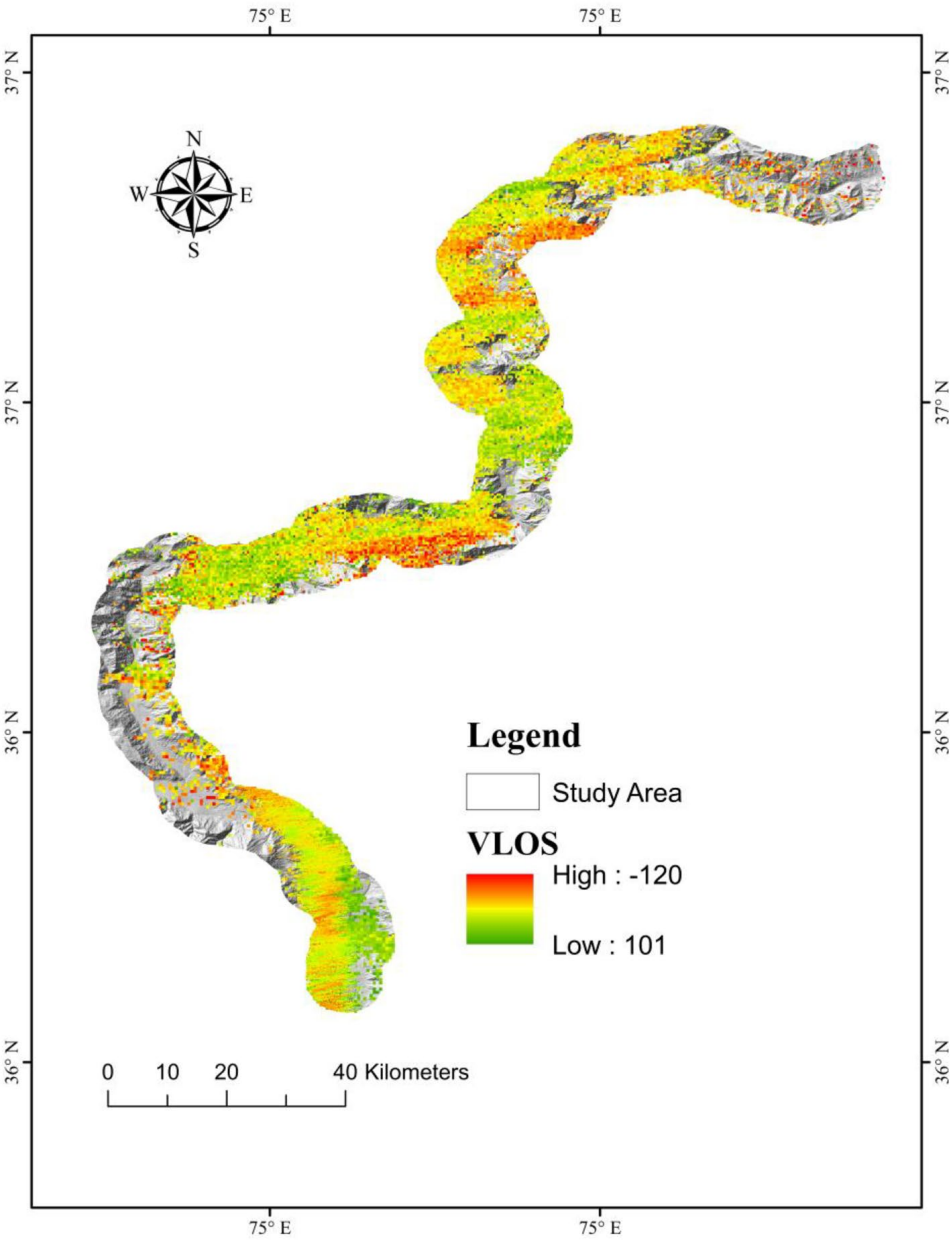
SBAS‐InSAR deformation velocity for landslide (VLOS) map across LOS direction for both ascending and descending data.

SBAS-InSAR methods were employed to evaluate and validate the models by verifying the deformation in the region. Due to its comprehensive high spatial-temporal resolution, it has the ability to operate and provides spatial coverage in all weather conditions. Over the past decade, the InSAR method for identifying and monitoring mass movement has become well-established^84^. To identify the ratio of slow-moving landslides, numerous SBAS-InSAR investigations have been carried out to evaluate the historical or spatial patterns of landslides distortion of slow-moving landslides^85^. In SBAS-InSAR processing, the line of sight (LOS) deformation velocity (VLos) was determined using 0.35 as the coherent threshold, as indicated in Table 3. Slope orientation velocity (Vslope) is determined using satellite line-of-sight (LOS) information. The Vslope shows the deformation only in a single direction. In landslide assertion, most landslides or the earth's surface displacements occur over steep terrain; consequently, Vslope is the key component used to estimate landslide development (Fig. 11). The regions on the map where SBAS-InSAR results show high deformation is also validated by XGBoost LSM (Fig. 9). According to the SBAS-InSAR results, the majority of marked landslides were observed to be deforming regions. Because of the extended re-visiting time of the Sentinel 1A sensor, slow-moving landslides may predict more accurately.

**Figure 12:**
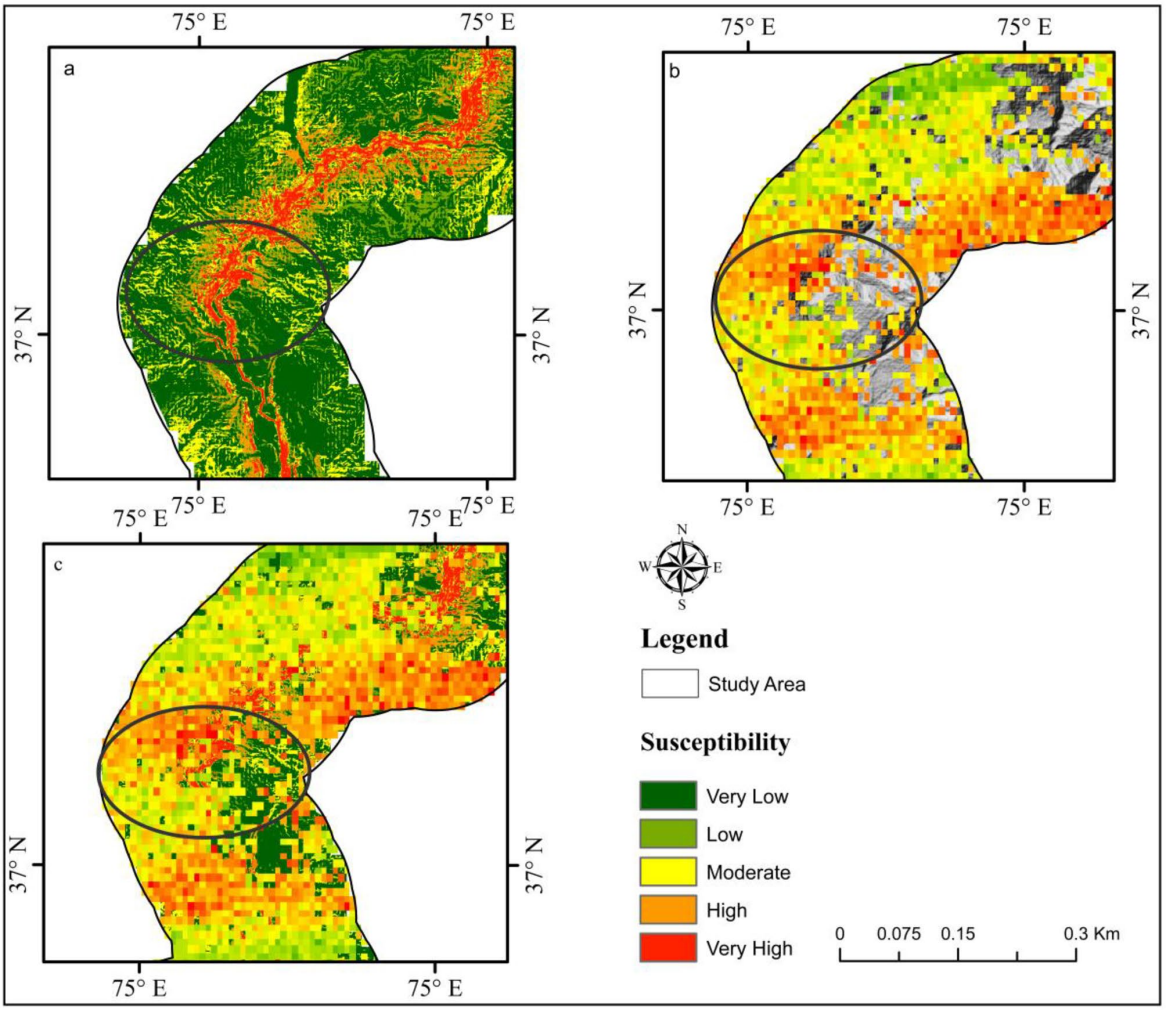
Landslide susceptibility Outcomes Upper Gojal area. (**a**) Using the XGBoost model, LSM outcomes were obtained. (**b**) SBAS‐ InSAR‐based landslide deformation velocity (Vslope) map. (**c**) Enhanced landslide susceptibility map result.

Figure 12 depicts a region with a notable rise in landslide vulnerability. Now we are able to give a comprehensive analysis of the location. The location is a steep region in the upper Gojal district of Hunza, primarily composed of loose Quaternary sediments. Both the wind and the rain have an important impact on them. The majority of the slope's steepness is less than 30°, making it inherently unstable. The soil's mechanical and physical characteristics are diminished due to the bank slope's gradual deterioration caused by long-term immersion in water. As the level of water fluctuates inversely and the water waves are eroded, the rocks and soil have grown less stable and steeper. At some stage, a certain level of local slip, destabilization and failure will occur. SBAS-InSAR displacement reveals a higher distortion rate, and an assessment of the probability of the landslide after SBAS-InSAR improvement confirms the improvement (Fig. 12).

**Figure 13:**
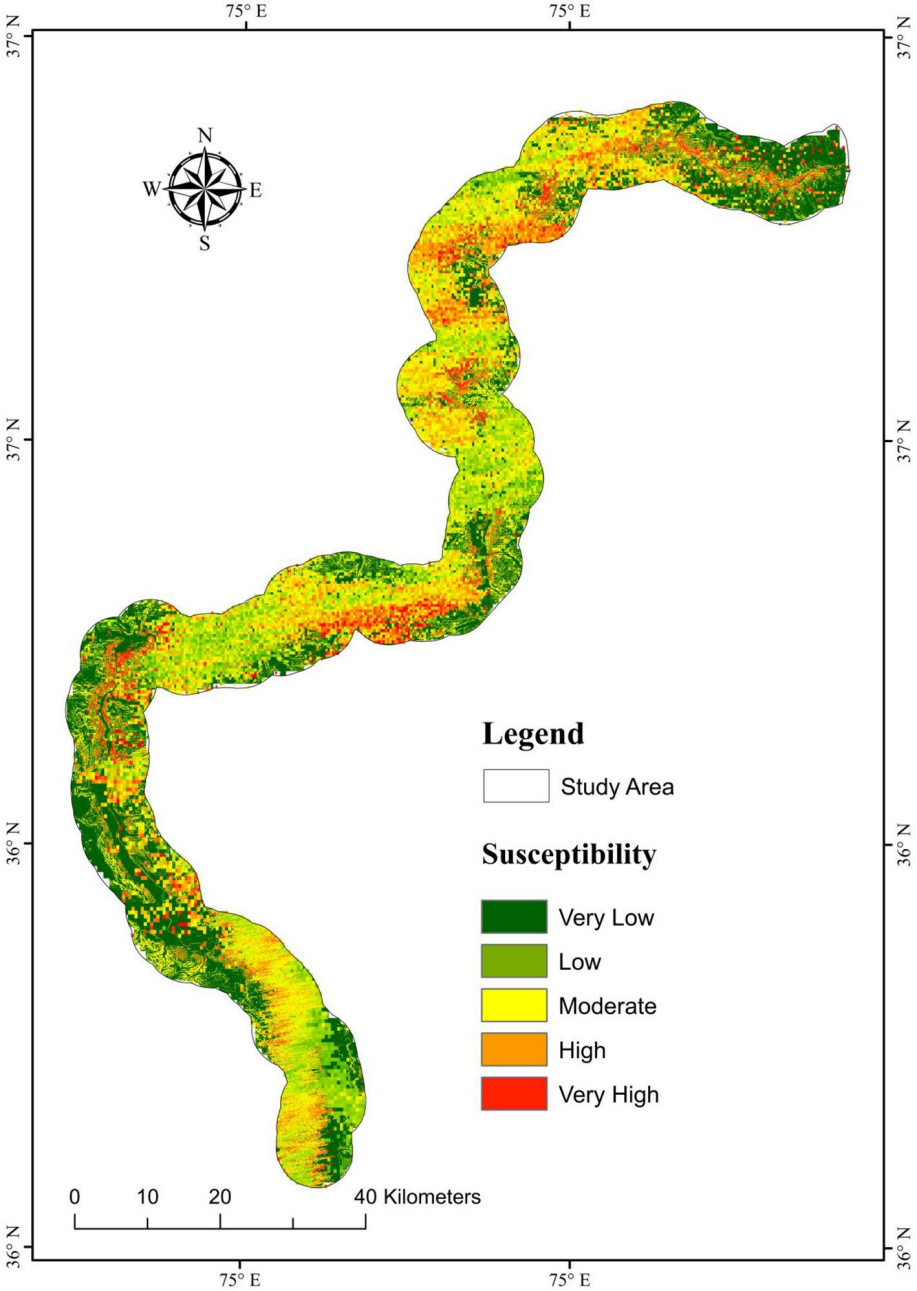
Through Vslope, the correction matrix was implemented to improve the model of landslide susceptibility.

Finally, the accurate deformation map for the vicinity was created by combining the Vslope and XGBoost-based LSM using the correction matrix (Fig.13). However, the newly developedsusceptibility map, created by the XGBoost model, was utilized to evaluate the amount of variation between each cell. The new map revealed that 10.67% of the research area is extremely prone to landslides, while values for high, moderate, low, and very low susceptibility classes were 11.34%, 22.81%, 28.64%, and 26.54%, respectively. However, the XGBoost model showed 5.54%, 6.52%, 13.28%, 13.24%, and 61.42%, respectively, for the regions with very high to very low susceptibility. Fig. 13 displays some regions where the probability of landslide susceptibility has significantly increased.

## Discussion

LSM is an important topic that supports risk management and planning in many areas globally^15^. The intricacy of the landslide hazard makes it more difficult to create accurate regional-scale maps in the mountainous region. Therefore, in resolving landslide-related engineering challenges, ML algorithms outperform more conventional methods. It is demonstrated that ML algorithms perform differently at different scales in diverse environments, depending on variables such as geology, climate, topography, and others^30^. Thus, using a single model for both mapping and modeling susceptibility is therefore unreliable. Therefore, it is essential to explore, analyze and understand the difference between the results of different ML algorithms to choose and identify the accurate model**.**

The outcomes demonstrate that the accuracy of the XGBoost, RF, NB, ANN, and KNN machine learning approaches for LSM along the KKH yielded satisfactory results. However, XGBoost surpassed the results of RF, NB, ANN, and KNN for evaluating LSM in terms of accuracy and AUC values, as described in Fig. 10 and Table 4. Comparatively, the XGboost model performed better in evaluating the significance of each factor in initiating landslides because of the optimal combination of processing time and prediction performance. The ability of XGBoost to predict LSM was demonstrated in previous research^6,86–90^. The performance AUC for XGBoost in this study is enhanced by selecting the most important LCFs and applying many trees leading to a good performance model.

Furthermore, the RF model achieved an AUC of 0.993 for evaluating LSM (Fig. 10). The findings of the RF reported in this research performed higher than those obtained in previous research to access susceptibility of landslides in Northern Vietnam and the Izu-Oshima in Japan,with reported AUC of 0.839 and 0.956, respectively^91,92^.

For the NB, the model yielded an AUC of 0.988, respectively. The outcomes revealed that the NB performed efficiently in evaluating LSM. The result revealed that the AUC and the accuracies of the NB are better than the results reported in previous investigations in China and Vietnam, which were 0.91 and 0.93, respectively^93^.

For ANN, The model's AUC in the prediction was 0.984, respectively. Also, the KNN model achieved an AUC of 0.924 in the prediction of LSM in this study. These findings outperformed previous studies with reported AUCs of 0.879 and 0.875 in the evaluation LSM in Kota Kinabalu, Malaysia^94^. The increased quantity of hidden units in the network training enhanced the performance of ANN in this study, as selecting more than one hidden layer improved the accuracy of the ANN model^95^. The ANN can be trained with an optimum number of two hidden layers for the network's training.

The statistical investigations demonstrate that the five ML models used in this study to predict the susceptibility of landslides achieved good results with high AUC values, indicating a high predictive power for LSM. The improved performance accuracies obtained in this research for the five models might be attributed to the relevant selection of LCFs. The findings indicated that among the five algorithms, the XGBoost had outperformed the other four ML models in predicting LSM for the research region (Table 4). The Variations in the different algorithm's predictive ability depend on the model's structure and the optimization parameters. The good prediction observed in the XGBoost model is attributed to the fact that the model does not focus on a single independent variable, due to which it achieved excellent results. Also, XGBoost is designed to train with multiple Processing cores, and it can identify and learn upon nonlinear data patterns, and regularized boosting is employed. Therefore the model can avoid the overfitting problem and enhances the prediction accuracy^15,96^.

The lesser performance obtained for the RF model compared to the XGBoost can be associated with the RF model's tendency to offer more preferences to hyperparameters in order to improve the model. Therefore a small change in the hyperparameter will influence the majority of trees in the RF, which can affect its prediction^97^. These issues can reduce the performance of the RF since XGBoost always prioritizes functional space while reducing the cost of a model, enhancing the model's performance. Also, the NB has lower performance than XGBoost and RF, which is caused by the fact that the NB cannot classify unbalanced datasets as effectively as XGBoost and RF

The lower performance of the ANN model relative to the XGBoost model can be attributed to the inability of ANN models to evaluate the training data. Therefore, overfitting is a difficult problem with ANN training data which can cause a lower model performance^98^. The KNN model has demonstrated the lowest performance among the employed models in this study (Table 4) and (Fig. 10). This lower performance results from the fact that the KNN may perform lower in high-dimensional data, leading to overfitting and inaccurate model. Regardless of KNN's lower performance in the current study, there are advantages to utilizing it to predict LSM in future research. The process of evaluating LSM is difficult to comprehend due to the existence of numerous environmental factors. However, the more adaptable the algorithm, the more efficient and accurate the model^15^. The performance of an algorithm depends on the algorithm data nature, structure, and selection of LCFs^15,92^.

**Figure 14:**
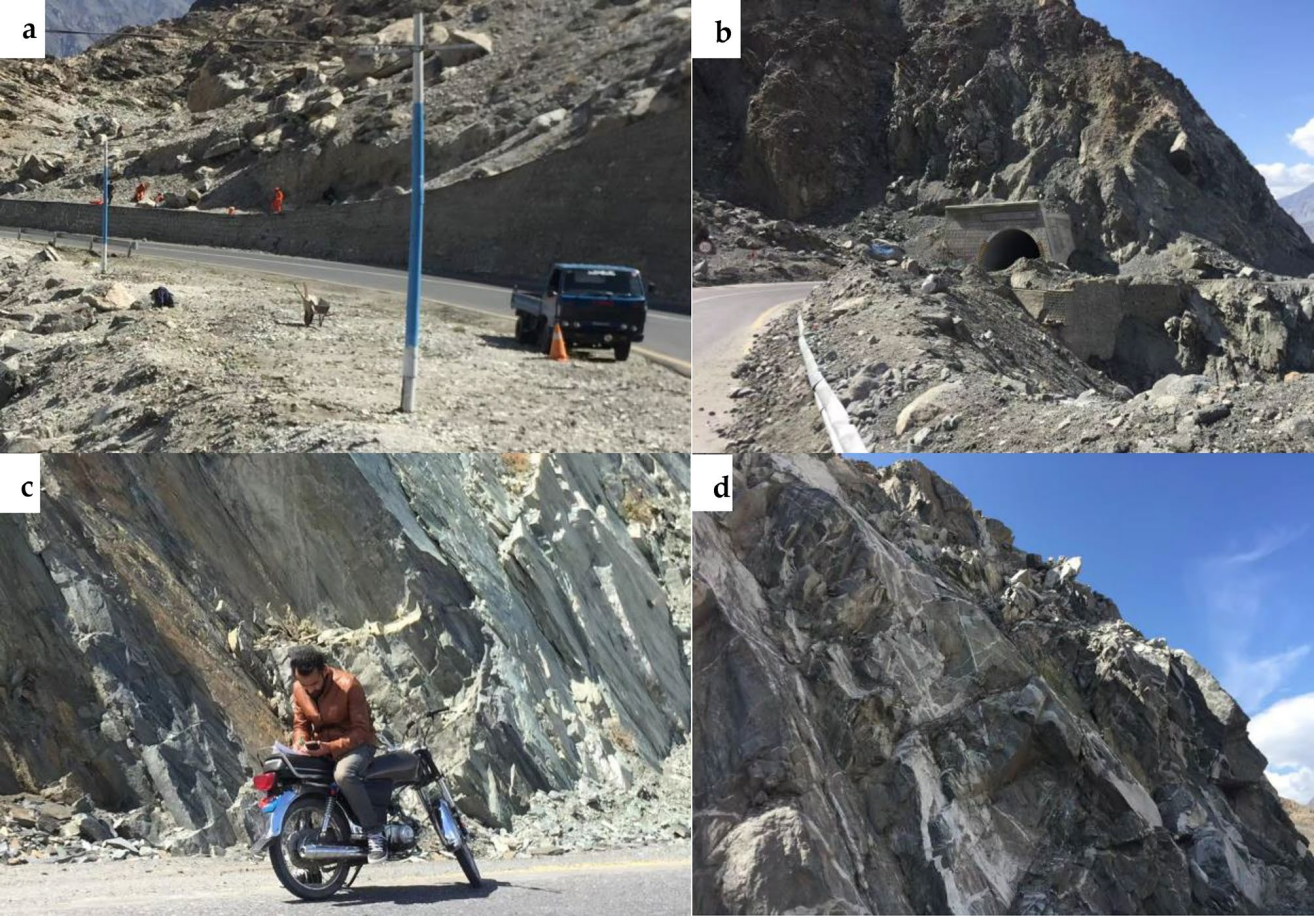
Investigation of various types of landslide during a field visit. (**a**) The Frontier Works Organization (FWO) clears the road after a rock fall in the Nagar District. (**b**) Debris flow in District Gilgit. (**c**) Using GPS to obtain actual landslide’s location. (**d**) Rockfall in District Hunza.

InSAR techniques can generate highly precise results, generating susceptibility maps with high-accuracy^99^. For this study, the SBAS-InSAR approach is applied to determine landslide displacement velocity and frequency in 2021. The landslide susceptibility map generated by combining XGBoost and SBAS-InSAR is categorized into five classes (Fig. 13). The XGBoost-SBAS-InSAR-based LSM displays that 10.67% of the total study region is highly vulnerable, demonstrating the model's accuracy. Using ML algorithms alone may lead to many constraints that can lead to misclassification when applied to conduct LSM. The first problem is related to the data quality of LCFs, whereas the second concern is past landslide history. Due to the hard conditions and environment of the research region, only 303 landslides over a 300-km length were mapped (Fig. 14), which may not accurately show the entire number of previous landslides. Thus, this can cause a significant inaccurate misclassification of the LSM, which can be reduced by employing the SBAS-InSAR technique. Thus, the outcomes of XGBoost and SBAS-InSAR were combined to create a new and improved landslide susceptibility map (Fig. 13) for the region, which minimized the misclassifications of slope-affected terrains. An issue with the LSM is that it predicts landslide occurrence in specific regions, not the continuous deformation movements with the passage of time. In contrast, variations in the occurrence of landslides over time are an important factor for decision-makers to consider^100^. The upgraded LSM, combined with the SBAS result in Fig. 13, provides landslide activity status for regional investigation and, at the provincial level, quantitative hazard assessment and mapping^101^.

## Conclusions

This study examines the mapping of landslide hazards along 332 km of the Karakoram Highway in the rugged mountainous terrains of Gilgit Baltistan, Pakistan. Landslides, rockfalls and debris flows are common along the KKH, disrupting its normal operations. Due to these natural disasters, many people lost their lives and wealth. Due to the rugged topography, mapping landslides using traditional methods is thought to be a difficult task in mountainous terrains, so this work represents a new method of landslide mapping and forecasting in which modest remote sensing techniques, GIS tools and different ML models are used to generate the LSM along KKH which is validated by SBAS-InSAR technique. Various causative factors of landslide, i.e., slope, geology, precipitation, TWI, proximity to the road, land cover, proximity to fault, proximity to a stream, roughness, aspect, profile curvature, curvature, plan curvature, and elevation, were used to train ML models, i.e., RF, XGBoost, RF, NB, ANN, and KNN to generate LSM. The findings showed that the primary source of landslides in the region is proximity to the road, slope, TWI and roughness. Outstanding forecasting outcomes were achieved using ML algorithms and SBAS-InSAR methods. The improved and final susceptibility map shows that 10.67% of the study region is extremely vulnerable to landslides.

The high, moderate, low, and very low susceptibility categories were 11.34%, 22.81%, 28.64%, and 26.5%, respectively. This work has significant implications for enhancing LSM, particularly in regions where the SBAS technique is appropriate and accessible. This improved LSM can help disaster management, mitigation, and prevention along the KKH. It also requires geotechnical and other slope stabilization techniques to reduce the possibility of future landslide disasters in a given area. We conclude that our method can provide significant information on highway precautionary measures.

## Data Availability

The study's first and corresponding authors can provide the data upon request. Because a thesis is being prepared using these data, the data are not publicly available.
